# Relevance of tumor-infiltrating lymphocytes in breast cancer

**DOI:** 10.1186/s12916-015-0431-3

**Published:** 2015-08-24

**Authors:** Sathana Dushyanthen, Paul A. Beavis, Peter Savas, Zhi Ling Teo, Chenhao Zhou, Mariam Mansour, Phillip K. Darcy, Sherene Loi

**Affiliations:** 1 grid.1055.10000000403978434Division of Research and Cancer Medicine, Peter MacCallum Cancer Centre, East Melbourne, VIC Australia; 2 grid.1008.9000000012179088XSir Peter MacCallum Department of Oncology, The University of Melbourne, Parkville, VIC Australia; 3 grid.1055.10000000403978434Peter MacCallum Cancer Centre, East Melbourne, VIC Australia

**Keywords:** Breast cancer, HER2, immunity, immunotherapy, NAC, neoadjuvant chemotherapy, TILs, TNBC, triple negative breast cancer, tumor-infiltrating lymphocytes

## Abstract

While breast cancer has not been considered a cancer amenable to immunotherapeutic approaches, recent studies have demonstrated evidence of significant immune cell infiltration via tumor-infiltrating lymphocytes in a subset of patient tumors. In this review we present the current evidence highlighting the clinical relevance and utility of tumor-infiltrating lymphocytes in breast cancer. Retrospective and prospective studies have shown that the presence of tumor-infiltrating lymphocytes is a prognostic marker for higher responses to neoadjuvant chemotherapy and better survival, particularly in triple negative and HER2-positive early breast cancer. Further work is required to determine the immune subsets important in this response and to discover ways of encouraging immune infiltrate in tumor-infiltrating lymphocytes-negative patients.

## Introduction

In cancer, neoplastic transformation alters the structure of tissues and induces immune responses leading to the elimination of developing tumors. However, incomplete elimination of transformed cells results in escape from immune control. This process is known as cancer immunoediting and is supported by a large body of experimental data and clinical evidence showing that the intact immune system can prevent and control cancer through the generation of effective tumor-specific immune responses [1, 2]. Immunoediting describes the process of malignant progression on the basis of tumor and immune cell interactions in three phases: (1) elimination, where cancerous cells are eliminated following immunosurveillance; (2) equilibrium, where transformed cells are held in control but are not eliminated by the immune system; and (3) escape, where tumor cell modifications shape disease progression [1, 2]. In general, a patient will present once the tumor has ‘evolved’ to escape immunosurveillance and, accordingly, a subset of patients with breast cancer present clear evidence of immune suppression and aggressive disease progression, potentially driven by mechanisms of tumor tolerance [3, 4]. In the elimination phase, the innate and adaptive immune system coordinate to detect and destroy cancer cells before clinical presentation. At this stage the balance is towards antitumor immunity stimulated by natural killer (NK) cells, NK-T cells, T cells, and increased pro-immune factors in the tumor microenvironment [2]. In equilibrium, there is a balance between antitumor and tumor-promoting factors, thus maintaining the tumor in a functionally dormant state [2]. Well-documented escape mechanisms of breast cancer cells include decreased immune recognition through reduced expression of major histocompatibility complex class I (MHC I) and/or co-stimulatory molecules and increased expression of immunosuppressive factors. This results in reduced clearance (lysis) via CD8^+^ cytotoxic T lymphocytes (CTLs) [3, 4]. The mechanisms underlying these processes have previously been reviewed in detail in several papers [1, 2, 5–10].

**Fig. 1:**
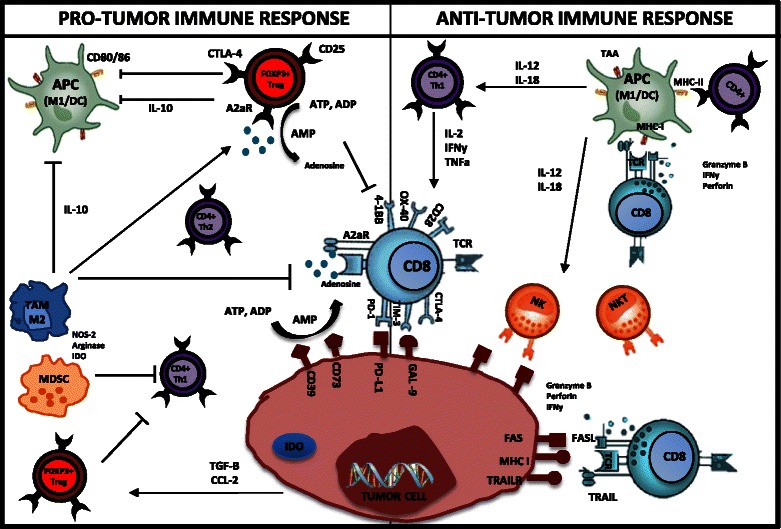
Interactions between the immune microenvironment and tumor cells in breast cancer. The antitumor immune response is dependent upon CD4^+^ (Th1) IFNγ production, which in turn mediates the expansion, differentiation, and activation of tumor-specific CD8^+^. CD8+ cytotoxic T cells induce cell lysis via recognition of specific TAAs such as MHC, FAS, and TRAILR on the surface of cancer cells/APCs. Similarly, CD4+ T cells are able to recognize MHC II on APCs. As a result of this complex formation (TCR-MHC/Peptide), high levels of granzymes, IFNγ, and perforin are released from CTLs, resulting in granule exocytosis and tumor cell death via apoptosis. NK and NKT cells with the help of APCs (DCs/M1) and CD4+Th1 are able to recognize and eliminate tumor cells. In the pro-tumor environment, CTLA-4, TIM-3, and PD-1 deliver inhibitory signals as a result of T-cell exhaustion/anergy caused by prolonged activation. CTLA-4 negatively regulates T-cell activation during the ‘priming’ phase of T-cell response. PD-1 expressed on T cells in the effector phase of T-cell response binds to its ligand PD-L1, expressed within the tumor microenvironment. This results in inhibition of T-cell activity (apoptosis). FOXP3+ Treg cells play a critical role during the selection of high-avidity CD8^+^ T cells, reducing their functionality. Tregs also have inhibitory action on APCs, CD8+ T cells, NKs, and CD4+ Th1 T cells. Both Tregs and tumor cells produce adenosine, which has inhibitory effects on T cells. Tumor cells can secrete cytokines and chemokines (e.g., TGF-β, CCL2) that recruit and stimulate suppressive cells such as Tregs, MDSCs, and M2 macrophages. M2 macrophages and MDSCs inhibit T-cell responses through nutrient sequestration via arginase, ROS, and NOS generation, as well as interference with trafficking into the tumor site. The upregulation of immunosuppressive enzymes such as IDO and arginase catabolizes essential nutrients required for effector cell activation. Furthermore, tumor cells downregulate MHC molecules, lose expression of antigenic molecules, and upregulate inhibitory molecules such as PD-L1, causing immune recognition to be inhibited and thus allowing immune escape and cancer progression. This figure was made exclusively for this manuscript. *A2aR* A2A adenosine receptor, *ADP* adenosine diphosphate, *AMP* adenosine monophosphate, *APC* antigen-presenting cell, *ATP* adenosine triphosphate, *CCl-2* chemokine ligand-2, *CTL* cytotoxic T lymphocyte, *CTLA-4* cytotoxic t lymphocyte-associated protein, *DC* dendritic cell, *FAS* fatty-acid synthase, *GAL-9* galectin-9, *IDO* indolamine 2,3-dioxygenase, *IFNγ* interferon gamma, *IL* interleukin, *M1/M1 TAM* tumor-associated macrophage, *MDSC* myeloid-derived suppressor cell, *MHC* major histocompatibility complex, *NK* natural killer, *NKT* natural killer T cell, *NOS* nitric oxide synthase, *PD-1* programmed death, *ROS* reactive oxygen species, *TAA* tumor-associated antigen, *TCR* T-cell receptor, *TGF-β* transforming growth factor beta, *TNFα* tumor necrosis factor alpha, *TRAIL* TNF-related apoptosis-inducing ligand, *Treg* T regulatory cell

Several studies have indicated that in addition to T cells, macrophages, NK cells, and dendritic cells (DCs) also infiltrate tumor tissue in varying capacities [1, 2, 8, 10]. It is known that CD4^+^ T helper 1 (Th1) cells, CD8^+^ cytotoxic T cells, NK cells, M1 macrophages, and DCs are protective against tumor growth [11]. Conversely, CD4^+^ forkhead box P3 (FOXP3^+^), CD4^+^ Th2 cells, M2 macrophages, and myeloid-derived suppressor cells (MDSCs) promote tumor growth [11]. These subsets interact in numerous ways; some of these mechanisms of interaction are shown in Fig. 1. Accordingly, tumor cells are able to suppress tumor-infiltrating lymphocytes (TILs) through multiple mechanisms either through direct suppression of antitumor immune cells or recruitment and reactivation of immunosuppressive subsets. One such mechanism is the expression of PD-L1 on tumor cells, which interacts with PD-1^+^ CD8^+^ T cells and induces subsequent anergy/apoptosis, leading to inactivation or exhaustion of TILs in the tumor microenvironment. This process leads to diminishing host antitumor immune responses [12]. Checkpoint inhibitors such as CTLA-4 and PD-1 trigger inhibitory pathways which dampen T-cell activity when bound to their ligands (CD80/CD86 and PD-L1/PD-L2) [13]. Both PD-1 and CTLA-4 blockade have proven to be very effective in preclinical animal models of melanoma and some breast cancer models [14–17]. Interestingly, an increasing number of studies are revealing positive outcomes in the clinical setting to checkpoint blockade of PD-1/PD-L1 and CTLA-4 [18–20]. Other targets that are of great interest in the clinical setting are the emerging T cell Ig and mucin domain (TIM) superfamily and lymphocyte activation gene 3 (LAG-3), given that they have both been associated with the inhibition of lymphocyte activity as well as induction of lymphocyte anergy [11, 12]. Additionally, several other immunosuppressive factors and inhibitory metabolites, such as adenosine [21–25], FOXP3^+^ regulatory T cells (Tregs) [26], indoleamine 2,3-dioxygenase (IDO) [27–30], arginase [31–33], and MDSCs [34–36], have been implicated in cancer-mediated immunosuppression, where targeting of these pathways has been shown to enhance antitumor immunity in vivo.

### Preclinical evidence for the role of the immune system in cancer and in response to chemotherapy and radiotherapy

Chemotherapy and radiotherapy are frontline management options for breast cancer and the underlying immunogenic component of these agents make them attractive candidates for neoadjuvant therapy. Preclinical studies of chemotherapy and radiotherapy have revealed the unexpected ability of the immune system to contribute to the success of treatment. There is an abundance of experimental and, more recently, clinical evidence [37–41] showing that chemotherapies are more efficient if they successfully re-activate immune surveillance through the elimination of immunosuppressive cells or through the promotion of danger signals released by the death of tumor cells, hence triggering a long-term immune response against residual tumor cells [42, 43]. Several findings have delineated a sequential series of events driving immunogenic tumor cell death that results in the activation of innate and adaptive immune responses [44]. During immunogenic cell death, dying tumor cells release danger signals such as adenosine triphosphate (ATP) and high-mobility group protein B1 (HMGB1), which act to prime an antitumor immune response [45, 46]. In landmark studies by Zitvogel and colleagues, anthracycline therapy was less effective in mice deficient in P2X7 receptors, NLRP3, and caspase-1, while oxaliplatin was dependent on HMGB1-mediated activation of toll-like receptors (TLRs). The activation of antigen-presenting cells such as DCs by ATP (P2X7) or HMGB1 (TLR4) leads to enhanced production of IL-1β, and consequently activation of both innate and adaptive immune responses [23]. Interestingly, the observation of enhanced antitumor immunity following treatment extends to various chemotherapies, including gemcitabine [47–49], cyclophosphamide [50, 51], paclitaxel [52], and doxorubicin [53–55]. Induction of tumor cell death by chemotherapy (e.g., gemcitabine) also enhances tumor antigen cross-presentation [56]. Furthermore, certain chemotherapies have direct effects on immune cells themselves. For example, low dose cyclophosphamide selectively reduces the number of immunosuppressive Treg cells while sparing immune effector cells [51]. These findings are summarized in Table 1. Many preclinical studies have described immune-enhancing activity in response to chemotherapies [57]. However, it is evident that not all chemotherapies induce immunogenic cell death. As such, it is apparent that combinations with specific immune-inducing or immune-targeted inhibitors are necessary to promote tumor regression and immunogenic cell death in these cases. Thus, despite initial theories suggesting that breast cancer is not an immunogenic disease, recent studies have confirmed and consolidated understanding of the underlying immunological component in breast cancer, thus revealing promising targets and novel therapies for treatment of this disease.

**Table 1:** Effects of chemotherapy on tumor-infiltrating lymphocytes

Drug	Target	Immunological effects and model used	Reference
Gemcitabine	Nucleoside analogue, prevents DNA replication	Reduction in the number of MDSCs	[126]
		HER2/neu model; breast cancer	[127, 128]
		Mesothelioma and lung cancer models	[48]
		Reduction in MDSCs and Tregs when given with cyclophosphamide; CT26 colon carcinoma	[49]
		Reduction of Tregs in patients with pancreatic cancer	
Cyclophosphamide	DNA alkylation, cross-links DNA	Induction of immunogenic cell death, IFN-I mediated activation of dendritic cells; EG7 thymoma, B16F10 melanoma	[129]
		Selective depletion of Tregs in MMTV-neu mice (breast cancer)	[88]
		Selective depletion of Tregs in colon carcinoma model	[42]
		Selective depletion of Tregs in melanoma model	[130]
		Reduction in MDSCs and Tregs when given with gemcitabine; CT26 colon carcinoma	[48]
Paclitaxel	Inhibition of mitosis through tubulin targeting	Reduction in MDSC frequency and suppressive activity	[131]
		Reduction in Treg numbers and suppressive activity	[52, 132]
Anthracyclines	Multiple mechanisms	Induction of immunogenic cell death; thymoma model, CT26 colon carcinoma	[45]
		Differentiation of CD11b^+^ LY6C^+^ APCs; MCA205 fibrosarcoma	[133]
		Elimination of MDSCs; 4 T1 and EMT6 breast tumor cell lines	[134]
Oxaliplatin	Cross-links DNA	Induction of immunogenic cell death resulting in the activation of myeloid cells; thymoma/colon cancer model	[45, 135]
Cisplatin	Cross-links DNA	Induce the accumulation of CD11c^+^ inflammatory dendritic cells; lung/colon carcinoma models	[136]
Docetaxel	Inhibition of mitosis through tubulin targeting	Reduction in MDSC frequency and suppressive activity; B16 melanoma model	[116]
5-aza-2′-deoxycytidine	DNA methyltransferase inhibition	Increased antigen presentation by tumor cells; 4 T1 breast cancer model	[137]
		Reduction in MDSCs and suppressive function; lung/prostrate carcinoma	[138]

*APCs* antigen-presenting cell, *MDSC* myeloid-derived suppressor cells, *Tregs* regulatory T cells

### Observations on the prognostic value of TILs in breast cancer

The importance of the immune system in breast cancer is increasingly being recognized, owing to the observation by several groups that the presence of TILs is a prognostic indicator for higher rates of pathological complete responses (pCRs) to neoadjuvant chemotherapy (NAC) [37, 58–60]. Notably, Denkert and colleagues [41] were able to first show in a large-scale analysis of 1058 patients’ biopsies that TIL^+^ tumors achieved a pCR rate of 40–42 % following NAC, whereas TIL^−^ tumors achieved a pCR of only 3–7 %. This distinction was based upon comparing lymphocyte predominant breast cancer (LPBC; defined as ≥60 % TILs in either the intratumoral or stromal compartment) to the rest of the population. Interestingly, this analysis revealed that TILs within either the stromal or intratumoral compartment was of value as a prognostic indicator. Subsequently, this has also been shown in another dataset of 580 triple negative breast cancer (TNBC) and HER2-positive breast cancers (59.9 % in LPBC vs 33.8 % in non-LPBC) [41] as well as in a meta-analysis of 996 patients where an immune gene module indicative of a Th1 response was prognostic of pCR in all cancer subtypes [61]. The protective role of a Th1 response was also reported by an independent group who found that the infiltration of T-bet^+^ lymphocytes correlated with a favorable prognosis [62]. TILs have been reported to predict pCR in a prospective study of HER2-negative breast cancers in patients treated with NAC, confirming the prognostic value of TILs in the response to chemotherapy [63].

**Fig. 2:**
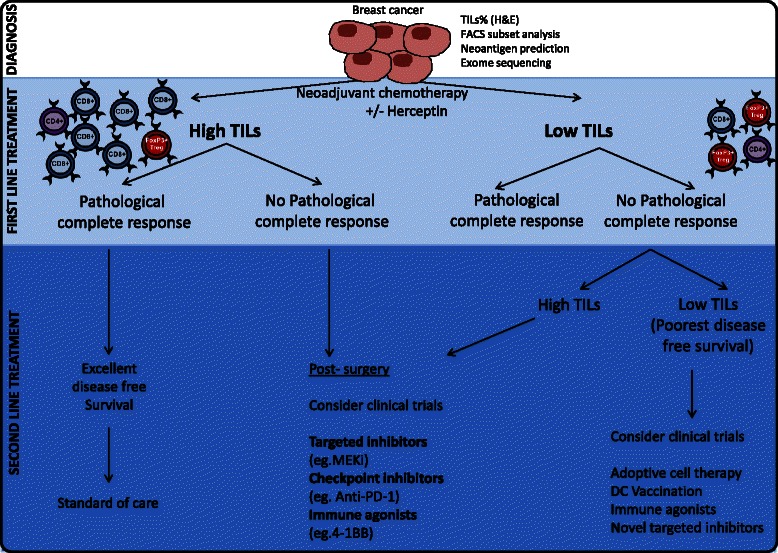
Using the TIL infiltrate and response to frontline treatments to guide patient management decisions. The presence of tumor-infiltrating lymphocytes (*TILs*) and response to neoadjuvant chemotherapy (NAC) may be used to guide decisions on second line treatments. Patients with high TILs and exhibiting pathological complete responses to NAC (*far left*) have an excellent prognosis and may not require further intervention other than standard of care. Patients with high TILs at diagnosis but no pathological complete response, or patients with low TILs at diagnosis but high TILs post-NAC, may benefit from immunotherapies, such as checkpoint inhibition (PD-1 blockade), or immune agonists (e.g., 4-1BB). However, patients with little TIL infiltrate either pre-NAC or post-NAC (*far right*) require additional or different treatment strategies to induce an immune response, such as adoptive cellular therapy or vaccination strategies. Targeted inhibitors (e.g., MEK inhibitors) should be considered for all patient groups where appropriate, but the impact of targeted inhibitors on the immune response should be a therapeutic consideration. This figure was made exclusively for this manuscript. *DC* dendritic cells, *FACS* fluorescence-activated cell sorting, *H&E* hematoxylin and eosin staining

From a clinical perspective, the observation suggests that higher levels of TILs are associated with higher responses to chemotherapy, seemingly independent of oestrogen receptor (ER), progesterone receptor (PR), and HER2. There is in fact no biologically appropriate TILs cut-point as all studies have shown that the TIL marker is prognostic on a continuous scale: each 1 % or decile increment is associated with a further increase in the rate of pCR. The biological reasons for this observation of chemosensitivity remain poorly understood. Perhaps pre-existing immune antitumor responses are better placed to be able to clear tumor cells after chemotherapy has depleted local immunosuppression or Treg cells. An increase in TILs in the breast cancer post-NAC is also associated with improved outcomes [64]. Notably, however, although TILs correlate with pCR to NAC in all breast cancer subtypes, the correlation between TILs at diagnosis and disease-free or overall survival was only significant in TNBC and HER2-positive breast cancers, although the reasons for this are not fully understood [39, 64–66]. The studies indicating a positive relationship between TILs and responses to NAC are summarized in Table 2. TILs are important in the efficacy of trastuzumab [65, 67, 68] and, moreover, trastuzumab treatment results in the activation or recruitment of multiple immune cell lineages and increases the susceptibility of tumor cells to antibody dependent cytotoxicity (ADCC) [67, 69] (reviewed by [70]). A study looking at TILs at baseline and correlation with both pCR and disease-free endpoints highlights that high levels of TILs at diagnosis bodes for an improved outcome regardless of attainment of pCR [68]. Those with low levels of TILs and residual tumor at surgery post-NAC and anti-HER2 therapy had the worst outcomes of all, suggesting that it is this group that needs more effective antitumor and immune-enhancing strategies. The fact that TILs are highly associated with responses to chemotherapy and trastuzumab suggests that the presence of TILs, both pre-NAC and post-NAC, should be taken into account for patient treatment decisions—a possible schema for this is shown in Fig. 2. The potential synergistic immune effects between chemotherapy and immunotherapies also suggest that combination approaches in the neoadjuvant setting, prior to surgery, may also be beneficial.

**Table 2:** A summary of the studies investigating a correlation between the extent of TIL infiltrate and responses to neoadjuvant/adjuvant chemotherapy in breast cancer

Breast cancer subtype (Number of patients)	TILs predictive of pCR?	TILs predictive of disease-free or overall survival?	Reference	Chemotherapy used
All patients (56)	Yes	ND	[60]	Anthracycline/taxane or epirubicin, cyclophosphamide, and capecitabine
All patients (73)	Yes	ND	[139]	Anthracycline/taxane based
ER^+^/PR^+^ (659)	Yes	ND	[59]	Anthracycline/cyclophosphamide/taxane
ER^−^PR^−^ (266)	Yes	ND		
All patients (1334)		Yes (total or distant stromal CD8+)	[75]	Cyclophosphamide/methotrexate/fluorouracil
ER^+^ (911)		No (intratumoral or adjacent stroma CD8+)		
ER^−^ (485)		No (total CD8^+^)		
HER2^+^ (169)		Yes (total CD8^+^)		
HER2^−^ (1106)		No (total CD8^+^)		
		Yes (total CD8^+^)		
ER^−^ (268)	Yes	Yes	[38]	Fluorouracil/epirubicin/cyclophosphamide or docetaxel and docetaxel plus epirubicin
One cohort of 113, one of 255				
All patients (3403)		No	[140]	Adjuvant systemic therapy
ER^−^ (927)		Yes		
ER^+^ (2456)		No		
HER2^+^ (216)		No		
TNBC (535)		Yes		
All patients (180)	Yes	ND	[37]	Anthracycline/cyclophosphamide or cyclophosphamide/epirubicin/5-fluorouracil
TNBC (82)	Yes	ND		
HER2^+^ER^−^PR^−^ (42)	No	ND		
HER2^−^ER^+^/PR^+^ (46)	No	ND		
All patients (845)	Yes	ND	[61]	Meta-analysis: anthracycline with or without taxane-based NAC
ER^−^HER2^−^	Yes	ND		
HER2^+^ (116)	Yes	ND		
ER^+^Her2^−^	Yes	ND		
All patients (68)	Yes	ND	[141]	Anthracycline and/or taxane-based treatment
All patients (180)	Yes	ND	[71]	Paclitaxel then fluorouracil/epirubicin/cyclophosphamide
HER2^−^ (313)	Yes	ND	[63]	Anthracycline/taxane
All patients (2009)	ND	No	[39]	Doxorubicin followed by three cycles of cyclophosphamide/methotrexate/fluorouracil
ER^−^/HER2^−^ (1079)	ND	No		
HER2^+^ (297)	ND	No		
TNBC (256)	ND	Yes		
All patients (153)	Yes	ND	[72]	Anthracycline and/or taxane-based treatment
TNBC (38)	Yes			
Non-TNBC (115)	Yes (If CD8^+^ component analyzed), No if CD4^+^ analyzed			
All patients (175)	Yes	ND	[73]	Anthracycline and/or taxane-based treatment or herceptin+NAC
All patients (12439)	ND	ND	[142]	Cyclophosphamide/methotrexate/fluorouracil or epirubicin plus fluorouracil
ER^−^ (3591)	ND	Yes		
ER^+^ (8775)	ND	No		
ER^+^HER2^+^ (772)	ND	Yes		
All patients (934)	ND	No	[65]	Docetaxel or vinorelbine, followed by three cycles of fluorouracil/epirubicin/cyclophosphamide
ER^+^HER2^−^ (591)	ND	No		
HER2^+^ (209)	ND	No		
TNBC (134)	ND	Yes		
TNBC (278)	Yes	Yes	[64]	Anthracycline-based neoadjuvant or anthracycline/taxane
TNBC (47)	Yes	ND	[143]	Panitumumab plus anthracycline/taxane-based chemotherapy
TNBC (481)	ND	Yes	[66]	Doxorubicin plus cyclophosphamide and taxol/docetaxol
All patients (580)	Yes	ND	[41]	Anthracycline/taxane with or without carboplatin
TNBC (314)	Yes	ND		
HER2^+^ (266)	Yes			

*ER* estrogen receptor, *HER2* human epidermal growth factor receptor 2, *NAC* neoadjuvant chemotherapy, *ND* not determined, *PR* progesterone receptor, *TNBC* triple negative breast cancer

Recently, efforts have been made to sub-divide the immune infiltrate into lineage subsets to determine the prognostic value of each immune cell type. The presence of CD8^+^ cells in the tumor infiltrate prior to the onset of NAC predicted pCR in several studies [41, 71–75]. The presence of Tregs prior to NAC has also been shown to be a prognostic indicator of pCR [71–73, 76]. Although this seems somewhat counterintuitive given the suppressive role of Tregs, it is probably because FOXP3^+^ infiltrate is also significantly associated with CD8^+^ infiltrate. More informative is the observation that the presence of Tregs following NAC has a significant negative correlation with pCR and disease-free or overall survival [77–79]. Indeed, the ratio of CD8 to Tregs following NAC has been shown to be a strong predictor of clinical responses [78]. The increased CD8 to Treg ratio likely facilitates the acquisition of CD8^+^ T-cell effector functions such as Granzyme B expression, which is elevated post-NAC [78, 80]. In this study they found that the proportion of CD8^+^ lymphocytes remained stable pre-NAC and post-NAC whereas Tregs were significantly reduced in the post-NAC samples. Limited work has been done on the prognostic significance of other immune cell subtypes although the presence of CD4^+^ [72] and CD20^+^ [74] lymphocytes pre-NAC is also associated with pCR. Another study found that the presence of T follicular helper (T_FH_) cells, which function to attract and promote the formation of memory B cells, was associated with improved responses to chemotherapy in breast cancer. These T_FH_ cells localized to peritumoral tertiary lymphoid structures, indicating there may be localized orchestration of antitumor immune responses in certain breast cancers [81]. The link between TIL infiltrate and patient outcome has led to heightened interest in utilizing immune-modulating strategies for patients with breast cancer. Other immune subsets of interest include γδ-T cells and Th17 cells. γδ-T cells show positive correlations with CD4^+^ FOXP3^+^ T cells, and are associated with poor outcomes and advanced tumor stage, as well as low disease-free and overall survival in patients with breast cancer [82]. However, further work is needed given the apparent antitumor role of γδ-T cells in some settings [83, 84]. In a small study of 30 patients, Th17 cells negatively correlated with disease stage [85] but further work is needed to evaluate their effect on immune responses to NAC [86]. Accordingly, further work is needed to determine whether these subsets are potential targets for immunotherapy.

### Combining conventional treatment with immunotherapy

The strong prognostic significance of TILs in breast cancer opens up important questions for patient management. In particular, it is likely that patients with high TILs post-NAC can benefit from strategies designed to enhance the immune response against the tumor. As discussed above, NAC has the potential to increase the CD8^+^ to Treg ratio, which correlates with the likelihood of pCR [87, 78]. Interestingly, certain chemotherapies, including cyclophosphamide [42, 51, 88], used to treat patients in the cohort studied by Ladoire et al., have been shown to specifically reduce the number of Tregs in preclinical models. Thus, it is worth considering the immunological consequences of any NAC regime. For example, concurrent depletion of Tregs through the use of immunotherapies such as anti-CTLA-4 mAb [89] may enhance the efficacy of NAC in inducing antitumor immune responses. There may also be clinical benefit in depleting other immunosuppressive populations such as MDSCs or tumor-associated macrophages [16]. A histopathological examination of breast cancer tissue pre-NAC and post-NAC by DeNardo et al. revealed that the presence of CD68^+^ macrophages inversely correlated with CD8^+^ TIL following NAC. Furthermore, depletion of this subset enhanced chemotherapy in preclinical models of breast cancer [90]. A recent study showed that a monocyte/DC metagene analysis held similar predictive value to NAC as the T cell/NK cell module [91]. Therefore, further work evaluating this in humans is warranted although the heterogeneity of this population makes characterization more complex than the T-cell infiltrate.

Although NAC is capable of inducing CD8^+^ infiltrate within the tumor microenvironment, the efficacy of the antitumor immune responses is likely limited by the presence of immunosuppressive networks. For example, a recent retrospective analysis of 94 post-NAC biopsies demonstrated that PD-L1 expression correlates with TIL infiltrate and pCR [40], which is in agreement with studies from other groups evaluating PD-L1 mRNA expression [41, 92]. This is likely because PD-L1 is expressed following IFNγ stimulation and so is a surrogate marker of an immune response. It should be noted, however, that other groups have reported that PD-1 and PD-L1 expression are correlated with poor prognosis in patients with breast cancer [93–95]. Nevertheless, these studies consistently observed PD-L1 and PD-1 expression in a subset of breast tumors; because PD-L1 expression has previously been shown to be a marker for the efficacy of anti-PD-1 in other tumor types [96], it is possible that PD-L1 expression may be a predictive marker for patients likely to have good responses to a combination of NAC and anti-PD-1. It was also recently reported that expression of B7-H4, another member of the B7 family, is a marker of good prognosis in patients with breast cancer [97]. Further work is need to evaluate the potential to modulate B7-H4 because it has been reported to inhibit T-cell function [98, 99] and yet B7-H4 expression was reported to limit tumor growth in this study [97]. Immune checkpoint blockade with anti-PD-1 [100] and anti-CTLA-4 [101] has already been shown to enhance the efficacy of chemotherapy and radiotherapy [102] in preclinical models and phase I clinical trials [103], and so there is clear rationale to further evaluate these combinations in patients. Further work will investigate the potential of combining NAC with other immune modulators in breast cancer given that that these immunosuppressive pathways appear upregulated in TIL^+^ breast cancers [41].

The success of checkpoint inhibitors is highly dependent on the extent of a pre-existing immune response against the tumor, and the ability of immune-targeted therapies to re-stimulate/activate immune subsets. Because conventional therapies such as chemotherapy and radiotherapy result in the release of tumor-associated antigens (increased antigenicity) from the apoptotic tumor cells, the increased immunogenicity provides a promising target for immunotherapies [103]. These tumor-associated antigens, released from tumor cells after chemotherapy, are taken up by antigen-presenting cells or DCs and used to stimulate downstream effector T cells capable of recognizing and lysing tumor cells [103]. Accordingly, it is evident that subsequent immunotherapy is likely to be a beneficial endeavor, provided that the initial conventional therapy used is able to effectively “prime” tumor cells to express and present foreign tumor antigens. A study conducted by Twyman-Saint Victor et al. demonstrated that radiation is able to enhance the diversity of the T-cell receptor repertoire of intratumoral T cells. This process was found to underpin the synergy of radiotherapy and anti-PD-1/anti-CLTA-4 in patients with melanoma [103]. Notably, high-dose radiotherapy and high-dose chemotherapy can ablate immune function. Therefore, careful consideration must be given to the dosage and the timing of these therapies when combined with immunotherapy.

### Combining targeted inhibitors with immunotherapy

While immunotherapy is an attractive adjuvant therapy for patients presenting with high TIL pre-NAC or post-NAC, an alternative approach is required for the treatment of patients with poorly immunogenic cancer types and low-level TILs at diagnosis. Liu et al. recently used preclinical models of melanoma to investigate the immunogenic function of the MAPK pathway inhibitors trametinib and dabrafenib in combination with immunomodulatory antibodies, including PD-L1/PD-1 and CTLA-4. They reported the potential for synergy between these therapies [104]. The combination of trametinib with anti-PD-1 increased tumor-infiltrating CD8^+^ T cells in CT26 mouse colon carcinoma tumors, as well as downregulating immunosuppressive factors, upregulating HLA molecules, and increasing immune responses in the tumors yes [104]. Dabrafenib had no suppressive action on the function of CD4^+^ and CD8^+^ T cells, whereas trametinib had mild partial or transient inhibitory effects on T-cell proliferation fine [104]. This phenomenon is supported by findings of Callahan et al. demonstrating that RAF inhibitors in BRAF wild-type tumors caused hyperactivation of ERK signaling, thereby enhancing T-cell activation and signaling [105]. While targeted blockade of checkpoint inhibitors has proven to be effective, recent evidence suggests that agonists may also be crucial in TIL stimulation. CD8^+^ T-cell responses require T-cell receptor activation in addition to co-stimulation provided through ligation of tumor necrosis factor receptor family members 4-1BB (CD137) and OX40 (CD134). Studies using the OX40 agonist in combination with either anti-4-1BB, anti-PD-L1, anti-CTLA-4, and immunization in sarcoma, melanoma, hepatoma, and breast cancer models have shown significant survival benefit by boosting the T-cell response [106–109]. One hurdle to the application of immunotherapy in breast cancer is the apparent low immunogenicity of breast cancers, perhaps owing to the low mutation rate of breast cancer when compared to cancers such as melanoma, which show a high response rate to immunotherapy [110–112]. One approach is to use targeted inhibitors designed to induce immunogenicity in tumors. For example, Kim et al. proposed the notion of epigenetic modulation, suggesting that MHC I-related genes are downregulated through epigenetic silencing of tumor cells [16]. They investigated this by combining anti-PD-1 and anti-CTLA-4 antibodies with 5-azacytidine (a DNA methyltransferase inhibitor) and entinostat (a class I histone deacetylase inhibitor). This study revealed complete eradication of tumors in murine 4 T1 (breast) and CT26 (colorectal) models [16]. Similarly, studies investigating DC vaccination in melanoma have shown that combination therapy with PI3K inhibitors has significant pro-inflammatory effects via TLR ligands that support antitumor immunity [113]. The results of these studies suggest that combining targeted inhibitors with immunotherapy may be a promising option for clinical treatment of breast cancer for patients with high-level TILs. However, alternative therapeutic options are still required for patients with low-level TILs. Because patients with high TILs show increased response rates to NAC, one approach is to use strategies that enhance T-cell trafficking to the tumor site. Denkert et al. reported a correlation between CXCL9/CXCL13 expression and the number of TILs in patients with breast cancer [59]. Therefore, treatments that enhance the expression of these chemokines may enhance the efficacy of NAC. Alternatively, adoptive cell therapy (ACT) utilizing TILs or chimeric antigen receptor T cells has emerged as a promising regimen for the treatment of certain cancers, including melanoma [114–118], with recent reports of long-term remission in some patients [119]. In a study conducted by Koya et al., combined treatment of vemurafenib plus ACT of lymphocytes genetically modified with a T-cell receptor recognizing ovalbumin resulted in superior antitumor responses [120]. While absolute numbers of T cells infiltrating the tumor did not increase with vemurafenib treatment, they found that the combination with ACT increased the functionality of antigen-specific T lymphocytes [120]. The findings of these studies suggest that BRAF inhibitors may be effective in combination with ACT; however, evaluation of ACT in combination with other inhibitors is also necessary. The utility of this approach in breast cancer is yet to be determined. One possibility is the targeting of the HER2 antigen [121, 122]. Ishikawa et al. [123] investigated the effects of ACT in addition to anti-CTLA-4, and were able to show that the combination enhanced antitumor activity. Taken together, there is a large body of evidence demonstrating the potential utility of TIL-associated immunotherapies for breast cancer in the future.

## Conclusions

There is now overwhelming data on the prognostic and predictive association of TILs. Determination of TILs is potentially universally available to all patients with breast cancer and efforts are underway to ensure that determination of TILs is standardized and reproducible [124]. If successful, the scene will be set for TILs to enter clinical practice as a biomarker that has the potential for clinical utility and prognostic implications [41].

Because TILs are associated with improved survival endpoints in a continuous fashion, they may be integrated with exiting models of risk prediction that inform decisions about adjuvant treatment, such as tumor size and nodal status, and may also serve as a stratification factor in randomized clinical trials. This may be of particular use in trial designs using NAC. pCR in the primary tumor with NAC is a very strong predictor of freedom from recurrence and long-term survival [125]. Of note, even patients without a pCR still have a relatively good outcome if they have high TILs in the primary disease [64, 124]. A composite of pCR and TILs could foreseeably be used to select patients who do not need further therapy because their risk of recurrence is negligible (pCR and high TILs) or low (pCR but low TILs), or conversely to select those at high risk of recurrence (no pCR, low TILs) who would benefit from trials of novel interventions that may include immunotherapy. Furthermore, using TILs to identify patients at very low risk of recurrence affords the opportunity for trials looking at de-escalation of therapy to avoid unnecessary long-term toxicity or focusing on novel therapeutic combinations for the poor prognostic group.
